# Quality of life in cervical cancer survivors after nerve-sparing radical hysterectomy, conventional radical hysterectomy or concurrent chemoradiotherapy

**DOI:** 10.3389/fonc.2025.1654730

**Published:** 2025-09-09

**Authors:** Wenhui Li, Xiuzhen Wang, Ming Wu, Xianjie Tan

**Affiliations:** ^1^ Department of Obstetrics and Gynecology, China-Japan Friendship Hospital, Beijing, China; ^2^ Department of Obstetrics and Gynecology, Peking Union Medical College Hospital, Peking Union Medical College & Chinese Academy of Medical Sciences, Beijing, China; ^3^ Department of Gynecology, Hainan General Hospital (Hainan Affiliate Hospital of Hainan Medical University), Haikou, China

**Keywords:** nerve-sparing, quality of life, anorectal dysfunction, sexual dysfunction, urinary dysfunction

## Abstract

**Background:**

Cervical cancer treatments, including radical hysterectomy and chemoradiotherapy, often lead to urinary, anorectal, and sexual dysfunctions. Nerve-sparing radical hysterectomy (NSRH) aims to reduce such complications, but evidence on long-term quality of life (QoL) remains limited.

**Objective:**

To compare QoL outcomes in cervical cancer survivors after NSRH, conventional radical hysterectomy (CRH), or concurrent chemoradiotherapy (CCR).

**Methods:**

A cross-sectional study enrolled 427 patients (241 NSRH, 60 CRH, 126 CCR) aged ≤60 years with ≥6 months post-treatment follow-up. QoL was assessed using EORTC QLQ-C30/CX24, Female Sexual Function Index (FSFI), and a self-reported questionnaire. Statistical analyses included ANOVA and chi-square tests.

**Results:**

Urinary symptoms occurred in 27.4% (NSRH), 40.0% (CRH), and 18.6% (CCR) (*p*=0.004). Anorectal symptoms were reported in 23.2% (NSRH), 25.0% (CRH), and 18.6% (CCR) (*p*=0.360). Among sexually active patients, FSFI total scores were higher in NSRH (23.8) vs. CRH (23.3) and CCR (21.6) (*p*=0.026). NSRH also showed superior sexual desire, arousal, and orgasm scores. QoL scores (EORTC QLQ-C30/CX24) indicated better outcomes in constipation (*p*=0.003), lymphedema (*p*<0.001), and sexual activity (*p*=0.027) for NSRH. Subgroup analysis confirmed NSRH alone had fewer complications than NSRH with adjuvant radiation.

**Conclusion:**

NSRH demonstrates significant advantages in preserving urinary, anorectal, and sexual functions, thereby improving QoL compared to CRH or CCR. It is a preferable option for early-stage cervical cancer, particularly in younger patients.

## Introduction

Cervical cancer (CC) remains a serious problem in developing countries (1, 2). Approximately 90% of cervical cancers occur in low- or middle-income countries as a result of inadequate screening and prevention.

Primary treatment options for cervical cancer could be radical hysterectomy (RH) or a concurrent chemoradiotherapy (CCR) regimen. But both have been reported with various complications. Vaginal, urinary, and bowel dysfunction are common sequelae, resulting from damage to autonomic nerves—specifically the sympathetic and parasympathetic branches that innervate pelvic organs and regulate their blood supply (3). Radiotherapy-related complications, including actinic cystitis, proctitis, and vaginal stenosis, have been reported in 23% of treated patients (4).

Quality-of-life outcomes have been receiving increasing attention (5). Japanese doctors modified the traditional radical surgery for CC to preserve nerve function. The main principle of this procedure is the preservation of both the inferior hypogastric plexus and the splanchnic nerves (6). After decades of practice, numerous studies have been published focusing on early postoperative complications, in particular spontaneous urinary voiding recovery (7, 8). It is widely believed that nerve-sparing modification significantly reduces urinary, colorectal, and sexual dysfunction. Unfortunately, data available on late morbidity are still inadequate. In this study, we carried out the investigation about late-term quality of life in cervical cancer survivors to assess the impact of various treatments and the value of nerve-sparing technique in radical surgery.

## Materials and methods

### Study subjects

Patients with primary cervical cancer who underwent radical hysterectomy or CCR ≥6 months prior were consecutively enrolled from the outpatient clinic of Peking Union Medical College Hospital (PUMCH) and China-Japan Friendship Hospital between February 2019 and February 2021. To be eligible for our study, patients needed to be under the age of 60 with no recurrent tumors discovered in the follow-up. Cases with mental illness or neurological disease were excluded from our study. Specifically, patients whose situations were combined with other malignancy and serious cardiovascular or respiratory diseases that affect their QoL were considered ineligible to participate. Moreover, participants who suffered from urinary, anorectal or sexual dysfunctions before treatment and those who could not use WeChat or had difficulty understanding the questions or filling out the questionnaires were also excluded from this study. Data on clinical characteristics including individual age, neoplasm staging and grade, histopathological type, and treatment methods was retrospectively retrieved from medical records. To explore the impact of different treatment methods on the quality of life of patients with cervical cancer, we divided them into three groups according to their initial treatment regimens, namely, nerve-sparing radical hysterectomy (NSRH) group, conventional radical hysterectomy (CRH) group and CCR group without surgery. This study was approved by the Institutional Review Board (IRB) of PUMCH, with the approval number JS-2020, dated June 25th, 2019.

### Assessment of physical function and quality of life

Multiple parameters were assessed using a 13-item self-reported questionnaire as well as the Female Sexual Function Index (FSFI), the European Organization for Research on Treatment of Cancer (EORTC) QLQ-C30 and its supplement QLQ-CX24 specific for cervical cancer patients for QoL evaluation focusing on three main areas of morbidity: sexual, bladder, and anorectal functions (9–13).

Bladder functions were characterized by the following parameters in the self-reported questionnaire: frequent urination, urinary urgency, urinary incontinence, straining to void, urinary retention, and increased nocturia, while anorectal functions were assessed by constipation, diarrhea, increased defecation frequency, fecal incontinence, exhaust incontinence, and exhaust mixed defecation. One question referring to the damage to quality of the patient’s sex life after treatment was also included in the questionnaire. To further evaluate the sexual life in patients enrolled, we used FSFI to show a more concrete appreciation, by 19 items assessing the 6 domains of sexual function: Desire, Arousal, Lubrication, Orgasm, Satisfaction, and Pain. The QLQ-C30 and QLQ-Cx24 were also used to evaluate QoL, containing 30 and 24 items respectively. QLQ-C30 consists of 5 function scales (Physical, Role, Emotional, Cognitive, and Social), 3 symptom scales (Fatigue, Nausea/emesis, and Pain), 6 single-item scales (Dyspnea, Sleep disturbance, Appetite loss, Constipation, Diarrhea, and Financial impact), and a Global QoL scale. In QLQ-CX24, the postulated scale structures were Symptom experience, Body image, Sexual/Vaginal functioning, and 5 single-item scales including Lymphedema, Peripheral neuropathy, Menopausal symptoms, Sexual worry, Sexual activity, and Sexual enjoyment. All scales and item scores were transformed to a 0–100 scale by the standard scoring algorithm, with a higher score representing a better level of functioning and a higher level of symptoms (14).

With over 570 million users, WeChat is considered to be the most popular social media platform in China. Several studies have shown the great potential and value of WeChat in healthcare work (15, 16). In this study, the questionnaire information entry were done in our cervical cancer patient database in advance, and the patients responded via scanning the two-dimensional code via WeChat. The submitted information was extracted from the database and managed by special researchers.

### Surgical technique

For early stage CC, radical hysterectomy is used as one of the most important primary therapies especially in young women. 10 years ago, the gynecological oncology research group led by Professor Ming Wu mostly used conventional radical hysterectomies (CRH) through a laparoscopic or open route to treat patients with early cervical cancer. Later, the technique was replaced by the nerve-sparing modification with the inferior hypogastric plexus and bladder branches being anatomically mapped and partially preserved to reduce the damage to their urinary, rectal and sexual functions (17, 18). Several hundreds of patients received this surgery, performed by the same group of doctors led by Professor Wu, and most received regular follow-up care in the outpatient department. The radicality of the NSRH is similar to the CRH procedure, and identical in the range of distal ureteral resection.

Our study compared outcomes following the NSRH to those of other therapeutic techniques.

### Statistical analysis

Data collection and analysis were performed using SPSS program version 24.0 (IBM). The indicators that described the occurrence of urinary, anorectal and sexual symptoms or dysfunctions were expressed with relative number composition ratios or rates (%). The categorical data were analyzed by a chi-square test, and the comparison of multiple groups of measurement data such as scores of questionnaires was performed by ANOVA. We employed the p-value of <0.05 to indicate statistical significance.

## Results

427 patients with a history of primary CC provided informed consent and took part in our investigation of their overall QoL focusing on the urinary, rectal, and sexual functions via WeChat. Women in our study aged from 28 to 59 years with a median age of 47 years. Out of all of the participants, 241 underwent NSRH, 60 underwent CRH, and 126 underwent CCR without surgery. Among the surgically treated patients, 236 of 241 in the NSRH cohort and all in the CRH cohort had the FIGO 2018 stage IA2–IIA disease; two patients were stage IIB, and staging data were unavailable for three. Patients allocated to the CCR group had advanced disease (IB1-IIIB) unsuitable for primary surgery. Because of the high risk factors from surgical pathology results that are associated with recurrence, 154 women (115 from the NSRH group and 39 from the CRH group) had to undergo further radiochemotherapy after the surgery.

All patients completed the self-reported questionnaire in the investigation to answer the questions about urinary and anorectal symptoms; 207 of 241 (85.9%) patients after NSRH, 54 of 60 (90%) after CRH, and 93 of 126 (73.8%) after CCR submitted the results of the FSFI. A total of 350 (82.0%) survey results regarding QoL based on QLQ-C30 and QLQ-Cx24 were collected, with 203 (84.2%) after NSRH, 45 (75%) after CRH, and 102 (81.0%) after CCR. Clinical characteristics mentioned above of all participants are summarized in **Table 1**.

**Table 1:** Characteristic of all patients enrolled.

Variable	NSRH	CRH	CCR	*p*
No. of patients	241	60	126	
Age at follow-up (years, median (range))	46 (28-59)	47.5 (36-57)	51 (30-59)	<0.001
Follow-up (months, median (range))	38 (6-143)	116.5 (9-185)	24.5 (6-153.5)	<0.001
Ovarian preservation	129 (53.5%)	42 (70.0%)	–	
Postoperative radiation	126 (52.3%)	39 (65.0%)	–	
Urinary symptom	66 (27.4%)	24 (40.0%)	22 (18.6%)	0.004
Anorectal symptom	56 (23.2%)	15 (25.0%)	22 (18.6%)	0.360
Sexual dysfunction	23/138 (16.7%)	6/30 (20.0%)	9/53 (17.0%)	0.935

NSRH, nerve-sparing radical hysterectomy; CRH, conventional radical hysterectomy; CCR, concurrent chemoradiotherapy.

### Urinary functions

All 427 patients with CC completed the self-reported questionnaire focusing on problems of urinary and rectal functions. Among the 6 questions associated with urinary function referring to frequent urination, urinary urgency, urinary incontinence, straining to void, urinary retention, and increased nocturia, the most common positive response was straining to void, which was reported in 9.0% of all cases. A total of 112 (26.2%) patients reported having at least one problem emerging or deteriorating after treatment, including 66 (27.4%) in the NSRH group, 24 (40.0%) in the CRH group and 22 (18.6%) in the CCR group. In the 3 groups of respondents, the incidence of urinary symptoms had a statistical difference, with a p of 0.004, and women were clearly less prone to urinary complications when nerve function was spared in the radical surgery (**Table 1**).

### Anorectal functions

We assessed the anorectal functions of 427 cases by asking about the parameters of constipation, diarrhea, increased defecation frequency, fecal incontinence, exhaust incontinence, and exhaust mixed defecation. The emerging or exacerbation of constipation at 11.4% was the most common complication among patients with CC during the follow-ups. In participants who underwent NSRH and reported complications of the anorectal system, 23.2% (56 cases) reported anorectal discomfort, which was less than the CRH group (25%) but slightly higher than the CCR group (18.6%) (p=0.360) (**Table 1**).

### Sexual functions

354 (82.9%) women from all completed the investigation of sexual functions by FSFI, and 218 (61.6%) were sexually active, which occupied 64.7% (134/207), 55.6% (30/54), 58.1% (54/93) in the NSRH, CRH and CCR groups, respectively. In patients who reported changes in their sex lives, 16.7% of cases in the NSRH group complained about deterioration, which was lower than both the CRH and the CCR groups slightly (p=0.935). The 19 items in FSFI covered five domains including Desire, Lubrication, Orgasm, Pain, and Satisfaction, and were scored on a 5-point Likert scale (12). Further analysis of the participants who were sexually active showed average overall FSFI scores to be 23.8, 23.3, and 21.6 in the NSRH, CRH, and CCR groups, respectively (p=0.026), suggesting the protection to vaginal function from nerve-sparing surgeries. Detailed scores are shown in **Table 2**. Participants experienced significant differences in sexual function according to various therapies in the FSFI total score (p=0.026) and FSFI subscale scores, including Desire (p=0.001), Arousal (p<0.001), and Orgasm (p=0.022). *Post hoc* analyses revealed significant improvements in sexual function with NSRH compared to both CRH and CCR.

**Table 2:** Scores of FSFI for sexually active patients.

Variable	NSRH	CRH	CCR	*p*
No. of patients	134	30	54	
FSFI: total score	23.8 ± 5.1	23.3 ± 7.0	21.6 ± 4.6	0.026
FSFI: Desire	3.2 ± 0.8	3.1 ± 0.9	2.7 ± 0.9	0.001
FSFI: Arousal	3.6 ± 1.1	3.5 ± 1.6	2.9 ± 0.9	<0.001
FSFI: Lubrication	4.2 ± 1.2	4.2 ± 1.1	4.1 ± 1.1	0.792
FSFI: Orgasm	4.2 ± 1.1	4.2 ± 1.5	3.7 ± 1.0	0.022
FSFI: Satisfaction	4.4 ± 1.1	4.4 ± 1.5	4.3 ± 1.1	0.834
FSFI: Pain	4.3 ± 1.3	3.8 ± 1.5	3.9 ± 1.4	0.170

Values are means ± SD. NSRH, nerve-sparing radical hysterectomy; CRH, conventional radical hysterectomy; CCR, concurrent chemoradiotherapy.

### Quality of life questionnaire

The QLQ-C30 and QLQ-CX24 were used in our study for further evaluation of participants’ QoL. As shown in **Table 3**, 203 (84.2%) cases in the NSRH group, 45 (75%) in the CRH group, and 102 (81.0%) in the CCR group completed the QoL questionnaire. Patients after NSRH obtained a higher score in many domains describing their QoL, but most scales didn’t show significant difference. Compared with conventional surgeries, the nerve-sparing technique significantly improved the conditions of Constipation, Lymphedema, and Sexual activity after treatment. Moreover, when assessing the domains of Symptom experience, Sexual activity, and even Financial difficulties, nerve-sparing surgeries showed obvious advantages, although we did not consider the effect of postoperative radiation.

**Table 3:** Scores of EORTC QLQ-C30 and QLQ-CX24.

Variable	NSRH	CRH	CCR	*p*
No. of patients	203	45	102	
QLQ-C30				
Physical functioning	84.8 ± 14.3	82.7 ± 16.9	83.3 ± 15.4	0.653
Role functioning	82.2 ± 22.1	82.2 ± 21.3	85.0 ± 21.7	0.572
Emotional functioning	77.6 ± 21.1	75.0 ± 22.9	76.7 ± 23.4	0.878
Cognitive functioning	78.9 ± 20.4	85.6 ± 13.9	77.9 ± 22.8	0.423
Social functioning	86.9 ± 18.4	81.1 ± 31.4	85.8 ± 21.3	0.542
Global QOl	79.3 ± 22.7	74.4 ± 27.9	78.3 ± 23.2	0.719
Fatigue	28.6 ± 23.0	31.8 ± 20.1	28.3 ± 19.9	0.843
Nausea/vomiting	5.7 ± 3.4	4.4 ± 3.9	6.7 ± 7.6	0.795
Pain	13.3 ± 12.4	8.9 ± 12.4	17.0 ± 20.2	0.125
Dyspnea	19.4 ± 14.8	13.3 ± 11.1	18.0 ± 12.9	0.602
Insomnia	31.7 ± 31.8	44.4 ± 32.5	36.9 ± 31.8	0.171
Appetite loss	10.5 ± 10.7	6.7 ± 10.8	15.7 ± 13.8	0.066
Constipation	24.0 ± 22.5	28.9 ± 25.3	13.4 ± 14.0	0.003
Diarrhea	14.0 ± 24.3	6.7 ± 13.8	17.6 ± 22.3	0.166
Financial difficulties	18.1 ± 16.6	15.6 ± 15.5	26.5 ± 26.7	0.028
QLQ-CX24				
Symptom experience	8.9 ± 7.3	8.1 ± 7.9	11.7 ± 8.8	0.024
Body image	14.5 ± 18.7	18.6 ± 23.5	14.7 ± 17.0	0.744
Sexual/vaginal functioning	27.2 ± 18.3	27.3 ± 13.5	28.3 ± 17.0	0.950
Lymphedema	25.3 ± 24.2	28.0 ± 24.9	9.4 ± 8.0	<0.001
Peripheral neuropathy	13.3 ± 18.8	22.8 ± 28.2	17.5 ± 21.7	0.112
Menopausal symptoms	26.1 ± 26.0	28.0 ± 29.9	31.4 ± 28.2	0.339
Sexual worry	26.7 ± 26.3	22.9 ± 31.5	31.0 ± 25.9	0.379
Sexual activity	21.6 ± 19.7	17.8 ± 21.8	14.7 ± 17.3	0.027
Sexual enjoyment	45.6 ± 26.2	49.7 ± 28.0	34.9 ± 21.3	0.082

Values are n (%) or means ± SD. NSRH, nerve-sparing radical hysterectomy; CRH, conventional radical hysterectomy; CCR, concurrent chemoradiotherapy.

### Subgroup analysis

To further clarify the benefits of nerve-sparing surgery in our group, it is necessary to divide the cases in NSRH to two subgroups: surgery alone (NSRH alone) or surgery plus adjuvant radiation (NSRH+R) (**Table 4**). As the statistical results summarized in the table show, cases after NSRH without postoperative radiation could experience less sexual or anorectal dysfunction as compared to the other two groups (p=0.973; p=0.016). Although urinary symptoms seemed more common in the NSRH group than the CCR group, but no difference were certified (p=0.085). In the evaluation of sexual function by FSFI, 188 patients reported active sexual lives during the follow-ups, and the mean total score of the NSRH alone group to be 24.6 was significantly higher than that of the CCR group and the combination treatment group of 21.6 and 23.0, respectively (p=0.004). In addition, the NSRH alone group had obviously superior scores in Desire, Arousal, Orgasm, and Pain when compared to the CCR group.

**Table 4:** Data obtained from subgroup analysis.

Variable	NSRH alone	NSRH+R	CCR	*p*
No. of patients	115	126	126	
Age at follow-up (years)	44.9 ± 6.6	45.3 ± 7.2	49.3 ± 6.9	<0.001
Follow-up (months)	34.7 ± 24.4	49.5 ± 28.5	24.1 ± 22.9	<0.001
Urinary symptom	27 (23.5%)	39 (31.0%)	22 (18.6%)	0.085
Anorectal symptom	18 (15.7%)	38 (30.2%)	22(18.6%)	0.016
Sexual dysfunction	11/69 (15.9%)	12/69 (17.4%)	9/53 (17.0%)	0.973
FSFI (sexually active)				
No. of patients	68	66	54	
FSFI: total score	24.6 ± 4.9	23.0 ± 5.3	21.6 ± 4.6	0.004
FSFI: Desire	3.3 ± 0.9	3.1 ± 0.7	2.7 ± 0.9	<0.001
FSFI: Arousal	3.6 ± 1.1	3.7 ± 1.1	2.9 ± 0.9	<0.001
FSFI: Lubrication	4.4 ± 1.1	4.0 ± 1.2	4.1± 1.1	0.117
FSFI: Orgasm	4.2 ± 1.0	4.2 ± 1.1	3.7± 1.0	0.021
FSFI: Satisfaction	4.5 ± 1.1	4.4 ± 1.1	4.3± 1.1	0.760
FSFI: Pain	4.8 ± 1.2	3.8 ± 1.3	3.9± 1.4	<0.001
QLQ-C30				
No. of patients	102	101	102	
Physical functioning	85.3 ± 14.9	84.2 ± 13.8	83.3 ± 15.4	0.618
Role functioning	81.2 ± 22.7	83.2 ± 21.5	85.0 ± 21.7	0.475
Emotional functioning	77.3 ± 21.4	77.9 ± 20.9	76.7 ± 23.4	0.931
Cognitive functioning	80.1 ± 20.8	77.7 ± 20.0	77.9 ± 22.8	0.686
Social functioning	86.4 ± 19.3	87.3 ± 17.5	85.8 ± 21.3	0.857
Global QOl	80.4 ± 22.4	78.1 ± 23.0	78.3 ± 23.2	0.737
Fatigue	29.8 ± 23.6	27.4 ± 22.5	28.3 ± 19.9	0.725
Nausea/vomiting	4.9 ± 11.6	6.6 ± 14.9	6.7 ± 17.6	0.627
Pain	15.2 ± 17.9	11.4 ± 16.8	17.0 ± 20.2	0.086
Dyspnea	19.3 ± 26.7	19.5 ± 22.7	18.0 ± 22.3	0.889
Insomnia	30.1 ± 30.2	33.3 ± 33.3	36.9 ± 31.8	0.306
Appetite loss	10.5 ± 19.3	10.6 ± 18.2	15.7 ± 23.8	0.118
Constipation	25.5 ± 22.4	22.4 ± 20.4	13.4 ± 13.0	0.003
Diarrhea	8.8 ± 7.5	19.1 ± 8.8	17.6 ± 12.3	0.003
Financial difficulties	13.7 ± 23.6	22.4 ± 28.7	26.5 ± 26.7	0.002
QLQ-CX24				
Symptom experience	7.8 ± 6.7	9.9 ± 7.8	11.7 ± 8.8	0.007
Body image	13.1 ± 17.9	15.9 ± 19.5	14.7 ± 17.0	0.595
Sexual/vaginal functioning	18.7 ± 13.0	34.1 ± 19.2	28.3 ± 17.0	<0.001
Lymphedema	24.8 ± 24.0	25.8 ± 24.4	9.4 ± 10.0	<0.001
Peripheral neuropathy	8.6 ± 5.5	17.8 ± 10.6	17.5 ± 11.7	0.003
Menopausal symptoms	21.7 ± 26.5	30.4 ± 24.9	31.4 ± 28.2	0.034
Sexual worry	18.9 ± 21.9	34.2 ± 28.0	31.0 ± 25.9	<0.001
Sexual activity	20.4 ± 20.5	22.8 ± 19.0	14.7 ± 17.3	0.020
Sexual enjoyment	50.7 ± 27.1	41.4 ± 24.9	34.9 ± 21.3	0.018

Values are n (%) or means ± SD. NSRH, nerve-sparing radical hysterectomy; R, radiation; CCR, concurrent chemoradiotherapy.

A total of 305 patients replied to the QoL questionnaires. Within these 3 groups, statistically significant differences were found regarding Constipation, Diarrhea, Financial difficulties, Symptom experience, Sexual/vaginal functioning, Lymphedema, Peripheral neuropathy, Menopausal symptoms, Sexual worry, Sexual activity, and Sexual enjoyment (p<0.05). The nerve-sparing technique improved patients’ QoL in all of the above domains except constipation and lymphedema as compared to CCR. Although there was no statistical difference between groups regarding other domains, we also found a tendency to have a better result about life quality in cases after NSRH.

## Discussion

Our study concentrated on assessing subjective symptoms that could directly affect CC survivors’ QoL. Data collection by WeChat from all participants after surgery or radiation gave our research a significant advantage, providing us with convenience and guaranteeing the privacy of respondents to some extent. As adjuvant radiotherapy was an important factor affecting the morbidity and thus could have biased the assessment of surgery outcomes, we further carried out subgroup analysis to eliminate the biases.

Urinary dysfunction was the most frequently reported late morbidity of radical surgery, with an incidence of 8%-80% in various studies assessed by subjective symptoms (19, 20) or using urodynamic technique (21, 22). As reported, symptoms could persist for 6 months or even longer in some patients and urinary incontinence, voiding by abdominal straining, and urinary retention were the most common symptoms (22, 23).

Over the past decade, NSRH has been adopted widely. A systematic review in 2018 reported a lower bladder dysfunction rates in the NSRH group compared with the CRH group (OR=0.39; 95%CI, 0.19-0.81), without a difference in survival outcomes (24). Urodynamic evaluation also concluded a similar result (25). In our research, the incidence of urinary symptoms in the NSRH group was 27.4% and it dropped to 23.5% after eliminating the effects of adjuvant radiation; these were clearly lower than such dysfunctions in the CRH group.

Few studies were reported to talk about changes of anorectal functions in CC survivors. A study including 531 women reported frequent dysfunctions such as severe straining (29%) and incomplete evacuation (26%) after RH (26). Assessed by anorectal manometry, an increased distention was required to trigger relaxation assessed, and internal sphincter relaxation and a decrease in rectal sensations happened in patients after RH (27). Constipation is the most widely published colorectal problem after CRH (28). The most common reaction to radiation may be the change of bowel habits such as urgency, diarrhea and tenesmus. Late radiation changes including endothelial damage, inflammation and fibrosis occurred more than 3 months after the completion of therapy, which led to the continuation of late-term anorectal or vaginal symptoms (29). We reported that 18.6% of patients completing radiochemotherapy experienced anorectal symptoms during the follow-up, which was higher than that of patients after NSRH without postoperative radiotherapy. Recent anatomical studies have demonstrated that autonomic innervation of the bladder and rectum follows distinct pathways, with bladder nerves running more laterally and rectal nerves being more medial and ventral. These anatomical differences may lead to different patterns of nerve vulnerability during radical hysterectomy, and could explain the variation in postoperative dysfunction (30).

Many studies on the changes of sexual functions in CC survivors have been published, but with controversial data. Most researchers believed CRH often caused changes of vaginal anatomy and function, consequently leading to changes in sexual health, such as dyspareunia, reduced sexual desire, and loss of orgasm (31). For patients who received CCR, a higher incidence of long-term sexual problems (70%) in comparison to surgery alone (5%-45%) were certified (32). Recently, favorable outcomes on recovery of vesical function, vaginal blood flow, and anorectal function associated with nerve preservation have been documented (33–35). Consistent with data from these available studies, we identified obvious improvements with the nerve-sparing surgery in comparison to the CRH and CCR groups. Patients reported fewer sexual symptoms and were assessed with better scores in several variables of FSFI or QLQ-C30/CX24.

Over the past ten years, our group has performed hundreds of NSRH on patients with early-stage CC. Thanks to the convenience of WeChat, we obtained and analyzed patients’ response to certain questionnaires, indicating obvious benefits of the nerve-sparing surgeries to their urinary, anorectal, and sexual functions as well as other aspects of QoL. We collected and analyzed data of patients treated by a same group of doctors to ensure the consistency of treatment level. And we restricted the patients to be under the age of 60 years and have a follow-up period over 6 months to decrease their influence to QoL. However, the sample size of patients after CRH was quite limited, which corresponds to an impaired test efficacy and an unstable result. We acknowledge that, owing to the real-world, retrospective design of this study, there were marked differences among the groups in sample size and in certain baseline variables. These discrepancies mainly arose from two factors: the evolution of clinical practice—NSRH has largely replaced CRH as the standard procedure at our institution, resulting in a limited CRH case load; and divergent treatment principles—surgery (NSRH/CRH) is reserved for early-stage disease, whereas CCRT is employed for locally advanced disease. However, by focusing on the very consequences of these initial treatment choices, we sought to compare the quality-of-life outcomes among the three cohorts. From this perspective, the groups remain comparable for the purposes of our research question. Restricted to unbalanced data collected in different cohorts. Most of the time, we merely compared the raw data of each dimension of quality of life under different treatment regimens, which to some extent demonstrated the benefits of neuro-protective surgery. However, we also understand the limitations of this study and hope to include more patients in the future. Through more rigorous grouping, we aim to reduce the impact of confounding factors on the credibility of the study. Moreover, some reported symptoms in the follow-up examination such as lymphedema are concerning, and many relevant factors have not been discussed in this article including adjuvant chemotherapy, educational level, and pre-treatment functional assessment, which could offer further explanations. Despite many studies confirming the oncologic results and prognosis of NSRH, additional prospective cohort studies are required. And we are carrying on a prospective research on prognosis and quality of life in cervical cancer patients for further validation and explanation.

## Conclusion

In our study, we identified that NSRH is encouraging as a treatment for young patients with early-stage CC because of their protection of urinary, anorectal and sexual function, and improvement of QoL, which could be a suitable option for initial treatment.

## Data Availability

The original contributions presented in the study are included in the article/supplementary material. Further inquiries can be directed to the corresponding authors.
